# Aggregation of Cationic Amphiphilic Block and Random Copoly(vinyl ether)s with Antimicrobial Activity

**DOI:** 10.3390/polym10010093

**Published:** 2018-01-19

**Authors:** Yukari Oda, Kazuma Yasuhara, Shokyoku Kanaoka, Takahiro Sato, Sadahito Aoshima, Kenichi Kuroda

**Affiliations:** 1 Department of Macromolecular Science, Graduate School of Science, Osaka University, Toyonaka, Osaka 560-0043, Japan; tsato@chem.sci.osaka-u.ac.jp; 2 Current, Department of Applied Chemistry, Kyushu University, Motooka, Nishi-ku, Fukuoka 819-0395, Japan; 3 Graduate School of Materials Science, Nara Institute of Science and Technology, Ikoma, Nara 630-0192, Japan; yasuhara@ms.naist.jp; 4 Current, Department of Materials Science, The University of Shiga Prefecture, Hikone, Shiga 522-8533, Japan; kanaoka.s@mat.usp.ac.jp; 5 Department of Biologic and Materials Science, School of Dentistry, University of Michigan, Ann Arbor, MI 48109, USA

**Keywords:** aggregation, amphiphilic block copolymer, poly(vinyl ether), antimicrobial activity

## Abstract

In this study, we investigated the aggregation behaviors of amphiphilic poly(vinyl ether)s with antimicrobial activity. We synthesized a di-block poly(vinyl ether), B38_26_, composed of cationic primary amine and hydrophobic isobutyl (*i*Bu) side chains, which previously showed antimicrobial activity against *Escherichia coli*. B38_26_ showed similar uptake behaviors as those for a hydrophobic fluorescent dye, 1,6-diphenyl-1,3,5-hexatriene, to counterpart polymers including homopolymer H44 and random copolymer R40_25_, indicating that the *i*Bu block does not form strong hydrophobic domains. The cryo-TEM observations also indicated that the polymer aggregate of B38_26_ appears to have low-density polymer chains without any defined microscopic structures. We speculate that B38_26_ formed large aggregates by liquid-liquid separation due to the weak association of polymer chains. The fluorescence microscopy images showed that B38_26_ bonds to *E. coli* cell surfaces, and these bacterial cells were stained by propidium iodide, indicating that the cell membranes were significantly damaged. The results suggest that block copolymers may provide a new platform to design and develop antimicrobial materials that can utilize assembled structures and properties.

## 1. Introduction

The emergence of drug-resistant bacteria poses a serious threat to human health [1,2,3], as the number of treatment options for bacterial infections is significantly reduced. There is urgent need for new antimicrobials effective in controlling drug-resistant bacteria. However, it has been a significant challenge to design and develop such molecules with novel antimicrobial targets in bacteria and mechanisms. To that end, one recent strategy is to design synthetic polymers to mimic the structural features and functions of host-defense antimicrobial peptides (AMPs) found in the innate immune system [4,5], which act directly by disrupting bacterial cell membranes. In general, antimicrobial (co)polymers have cationic and hydrophobic moieties in their side chains to mimic the cationic amphiphilicity of AMPs, which govern the bacterial selectivity and membrane-disrupting mechanism for antimicrobial activity [6,7]. The cationic groups of polymers enhance the binding of polymers to anionic lipids of bacterial membranes by electrostatic interactions. Because the bacterial membranes are more negatively charged than those of human cell membranes, the polymers are expected to selectively bind to bacterial membranes over human cell membranes, imparting the selective activity of polymers to bacteria over human cells. Upon the binding of polymers to membranes, the hydrophobic groups of polymers are inserted into the hydrophobic domain of the membranes, causing membrane disruption and ultimately bacterial cell death. It has been previously demonstrated that the antimicrobial activity of polymers and their toxicity to human cells can be controlled by modulating key structural parameters, including compositions of cationic and hydrophobic monomers [8,9,10,11], molecular weight [11,12], the hydrophobicity of side chains [13], and the type of cationic charge [14].

Synthetic polymers with cationic and hydrophobic segments or cationic amphiphilic block copolymers have been utilized as a platform for designing antibacterial polymers [15,16]. Such block copolymers are prepared by living polymerization, their length of polymer chains and block sequences can be precisely designed and controlled, which provides great advantages for the development of materials with target biological functions [17]. We previously synthesized a series of di-block poly(vinyl ether)s composed of cationic and hydrophobic blocks and investigated the relationship of their amphiphilic structures (block vs. random) with their antibacterial activity and lytic activity against human red blood cells (hemolysis) as a measure of undesired toxicity to human cells [15]. We demonstrated that the amphiphilic structures of these copolymers play an important role in their antibacterial and hemolytic activities [15]. The random and di-block copolymers with the same cationic/hydrophobic monomer compositions showed the same level of bactericidal activity against *Escherichia coli*. However, the block copolymers were not hemolytic, while the random copolymers were highly hemolytic. This result suggested that the block copolymers were selective to bacteria over human red blood cells while they remained active against bacteria, which is the desired properties for antimicrobials. A static light scattering (SLS) experiment suggested that the block copolymer formed aggregates with a diameter of ~500 nm in an aqueous media, which may be a vesicle rather than polymer micelles with a single hydrophobic core. Interestingly, the minimum polymer concentration of the block copolymer for bactericidal activity was below its critical (intermolecular) aggregation concentration (CAC), indicating that single-polymer chains were bactericidal. In addition, the copolymer was not hemolytic throughout the polymer concentration range above and below the CAC, suggesting that the selective activity of copolymer to bacteria over human cells was not necessarily the results of polymer aggregation or vesicle formation. We proposed the mechanism that the cationic polymer block wrapped the hydrophobic polymer block to form cationic single chain polymer particles. This particle structure shielded the hydrophobicity of copolymer chains and reduced their non-specific hydrophobic binding to the membranes of human red blood cells, resulting in no significant hemolytic activity [15]. On the other hand, the random copolymers might not be able to effectively shield the hydrophobicity of copolymers, because of the random distribution of cationic and hydrophobic groups in the polymer chains in comparison to block copolymers, and may thus bind to human red blood cells and cause hemolysis. It is generally known that there is an equilibrium between free single-polymer chains and aggregates above the CAC, and the concentration of single-polymer chains remains constant above the CAC. Our results indicate the possibility that single-polymer chains free in solution were responsible for the selective bactericidal activity of copolymer rather than the polymer aggregates.

In this study, we further extend our previous study on antimicrobial copolymers to investigate their aggregation behaviors in an aqueous environment. Amphiphilic copolymers intrinsically form aggregates and/or assemblies in aqueous media [18,19], which may control the interactions with bacterial cell membranes that govern the membrane-disrupting mechanism, thus determining the antimicrobial activity and selectivity. Therefore, it is important to investigate the formation and physicochemical properties of polymer aggregates in order to understand the role of aggregates in their underlying antimicrobial mechanism toward the goal of development of a novel class of antibacterial polymers. Specifically, the objective of this study is to determine the formation of polymer aggregates in water and their structures. In particular, we are interested in the aggregates formed by the block copolymer, because it previously showed potent bactericidal activity with selectivity to bacteria over human cells, which will be a good candidate for a new antimicrobial polymer platform. To that end, we first examined the uptakes of a hydrophobic probe by the copolymers to determine the formation of hydrophobic domains or polymer aggregates. The structure of block copolymer aggregates was further examined by a cryogenic transmission electron microscopy (cryo-TEM) that enables in situ visualization of the polymer assembly in water. The interaction between aggregates and bacterial cells was also examined by using fluorescent microscopy.

## 2. Materials and Methods 

### 2.1. Materials

All materials for polymerization were prepared and used as described in the previous report [15]. 4-(2-Hydroxyethyl)-1-piperazineethanesulfonic acid (HEPES) and fluorescein isothiocyanate (FITC) were purchased from Fischer Scientific (Waltham, MA, USA) and Sigma-Aldrich (St. Louis, MO, USA), respectively.

### 2.2. Synthesis of Amphiphilic Copolymers

**Figure 1:**
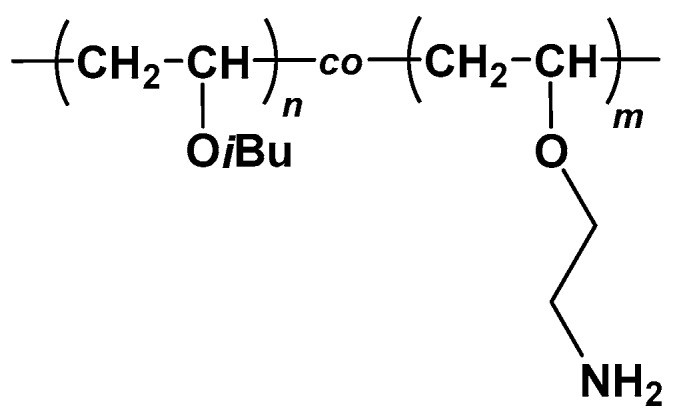
Chemical structure of poly(IBVE-*co*-AEVE)s.

A series of amphiphilic poly{(isobutyl vinyl ether)-*co*-(2-aminoethyl vinyl ether)}s {poly(IBVE-*co*-AEVE)s} (Figure 1) were prepared by living cationic copolymerization of IBVE and 2-phthalimidoethyl vinyl ether (PIVE), which was a protected monomer for AEVE, and subsequent deprotection as described in the previous report [15,20].

A FITC-labeled block copolymer was prepared by the reaction of the amino-containing block copolymer with FITC in the presence of trimethylamine in *N*,*N*-dimethylformamide at room temperature for 4 h, as described in the previous report [15]. The obtained FITC-labeled block copolymer was purified by size exclusion chromatography (Sephadex LH-20 gel, Amersham Bioscience, Uppsala, Sweden) using methanol.

### 2.3. Dye Uptake Experiment

The dye uptake by the polymer aggregates in the aqueous solution was examined using a fluorescent probe, 1,6-diphenyl-1,3,5-hexatriene (DPH) [21]. Polymer stock solutions were prepared in dimethyl sulfoxide (DMSO) (10 or 20 mg/mL). The stock solution was serially diluted 16 2-fold by 0.01% acetic acid. The polymer stock solutions (20 μL) were mixed with HEPES buffer (10 mM HEPES, 150 mM NaCl, pH 7, 175 μL) on a 96-well black microplate. DPH in tetrahydrofuran (THF) (20 μL, 50 μM) was diluted with HEPES buffer (480 μL). Then this DPH solution (5.0 μL) was added to the polymer solution on the microplate to give a final concentration of 50 nM for DPH, and THF of 0.1 vol %. After a 1 h incubation at 37 °C with orbital shaking (100 rpm), the fluorescence intensity in each well was recorded using a microplate reader (Thermo Scientific Varioskan Flash, Fischer Scientific, Waltham, MA, USA) with excitation and emission wavelengths of 357 and 430 nm, respectively.

### 2.4. Fluorescence Microscopic Observation

A single colony of *E. coli* was incubated in Mueller-Hinton (MH) broth at 37 °C with gentle shaking overnight. The *E. coli* suspension was diluted by MH broth to OD_600_ = 0.1 (OD_600_: optical density at 600 nm) and incubated again for 90 min. The bacterial culture in the midlogrithmic phase (OD_600_ ~ 0.5–0.6) was diluted to OD_600_ = 0.1 with HEPES buffer, corresponding to ~2 × 10^7^ cfu/mL (cfu: colony forming unit). This bacterial suspension (40 μL) was mixed with the stock polymer solution containing a small amount of FITC-labeled polymer (200 μg/mL, 50 μL) in a 96-well polypropylene microplate, which was not treated for tissue culture (Corning #3359). After a 45 min incubation at 37 °C, propidium iodide (PI) aqueous solution (16 μM, 10 μL) was added to the mixture and then incubated for additional 15 min. Confocal fluorescence microscopy images of the mixtures were recorded using Eclipse T*i* Confocal Microscope C1 (Nikon, Melville, NY, USA). FITC and PI were excited at 488 and 561 nm, respectively.

### 2.5. Cryo-TEM Observation

The specimen for cryo-TEM was prepared by rapid freezing of a polymer solution at a concentration of 10 mg/mL. A 200 mesh copper microgrid was used and pretreated with a glow-discharger (HDT-400, JEOL, Tokyo, Japan) to make the microgrid surface hydrophilic. An aliquot (3.0 µL) of a polymer sample was placed on the mesh and immediately plunged into liquid propane using a specimen preparation machine (EM CPC, Leica, Wetzlar, Germany). The temperature of the specimen was maintained below −140 °C during the observation using a cryo-transfer holder (Model 626.DH, Gatan, Pleasanton, CA, USA). Microscopic observations were carried out using a transmission electron microscope (JEM-3100FEF, JEOL, Tokyo, Japan) at an acceleration voltage of 300 kV in zero-loss imaging mode. The microscopic image was recorded using a CCD camera (Model 794, Gatan, Pleasanton, CA, USA) installed in the microscope.

## 3. Results and Discussion

### 3.1. Polymer Design, Synthesis, and Antimicrobial Activity

In this study, amphiphilic block (B38_26_) and random (R40_25_) poly(IBVE-*co*-AEVE)s with almost the same degree of polymerization (DP ~40) and compositions of hydrophobic IBVE (~25 mol %) were used. The synthesis and antimicrobial activities of these copolymers have been reported previously [15]. Briefly, the copolymers were synthesized by living cationic polymerization using protected monomer, PIVE, followed by removing the phthalate groups to give primary amine groups. The deprotected copolymers were denoted as R/BX_y_ (R: random, B: block, X: total DP, y: mol % of IBVE) using the values of protected polymers (Table 1). We also prepared a cationic homopolymer H44 for comparison.

These copolymers showed a bactericidal activity against *E. coli* [15]. The lowest polymer concentration to kill *E. coli* at least 99.9% of initial seeding concentration after 4-h incubation in HEPES buffer at 37 °C (BC_99.9_) was determined as a measure of the bactericidal activity of copolymers. We used a non-growth defined medium of HEPES buffer for our antimicrobial assay, as well as for characterization of the polymer aggregation. The BC_99.9_ values of B38_26_ and R40_25_ were very similar, indicating that the copolymer structures (random vs. block) do not determine the antimicrobial activity against *E. coli*. On the other hand, R40_25_ was highly hemolytic, showing a small HC_50_ value, while B38_26_ did not cause significant hemolysis (Table 1) [15]. Here, the HC_50_ values were defined as the polymer concentration required to cause 50% hemolysis relative to the positive control. 

### 3.2. Dye Uptakes by Copolymers

In the previous study, we determined the formation of aggregates of B38_26_ and R40_25_ by static and dynamic light scattering (SLS and DLS) [15]. We found that B38_26_ formed large spherical aggregates with a diameter of 400–500 nm above CAC of 36 μg/mL, whereas R40_25_ formed smaller aggregates with a diameter of 54 nm above CAC of 380 μg/mL (Table 1).

To further examine the role of hydrophobic side chains in copolymer aggregation, we first determined the critical aggregation concentration of polymers (C_DPH_) by monitoring uptake of a hydrophobic dye, DPH into the hydrophobic domains of formed polymer aggregates. The DPH probe has been widely used in the field to determine the critical aggregation concentrations of polymers, because its fluorescence property is sensitive to the polarity of the surrounding environment; the fluorescence of DPH increases upon partitioning into a non-polar or hydrophobic environment, while DPH in aqueous media is only slightly or not at all fluorescent [21]. The fluorescence intensity would increase when the polymer chains associate to form hydrophobic domains, and then take up the dye. Therefore, the DPH uptake would reflect the formation of microscopic hydrophobic domains due to association of hydrophobic side chains or block segments of polymers studied here. 

**Figure 2:**
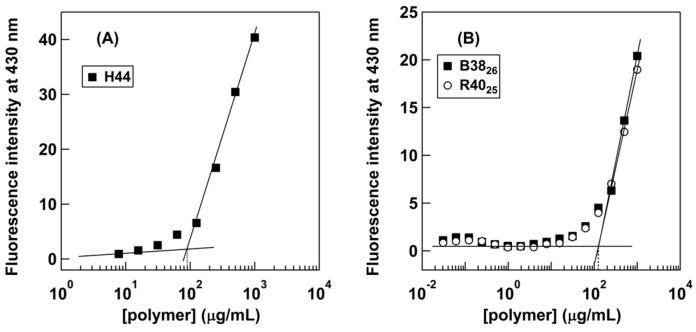
Fluorescence intensity of DPH (50 nM) versus polymer concentrations of (**A**) homopolymer, H44 and (**B**) poly(IBVE-*co*-AEVE)s with MP_IBVE_ ~25 mol % in HEPES buffer (pH 7). The data points represent the average from duplicate measurements.

All the polymers showed similar DPH uptake behaviors, resulting in the similar C_DPH_ values of 90–125 μg/mL (Table 1, Figure 2). This result indicates that the formation of aggregates of these polymers is not dependent on (1) the hydrophobicity of polymers (homopolymer vs. amphiphilic copolymers) and (2) copolymer amphiphilic structures (random vs. block copolymers). Other block and random copolymers with larger MP_IBVE_ values also showed similar DPH uptake behaviors (Table S1 and Figure S1), supporting the conclusion. 

Interestingly, the homopolymer H44 exhibited DPH uptake, although this polymer has no hydrophobic *i*Bu side chains. This result suggests that the cationic homopolymer can form hydrophobic domains and bind DPH molecules, likely as a result of their hydrophobic polymer backbones. Such hydrophobic domains can be formed by single polymer chains intramolecularly, or association of multiple polymer chains (intermolecular aggregation). Therefore, the C_DPH_ value may reflect either the onset of DPH binding curves by single polymer chains or the formation of intramolecular aggregates, but not necessarily formation of large polymer aggregates such as micelles. 

On the other hand, B38_26_ and R40_25_ also showed similar DPH uptake behaviors to H44, indicating that the hydrophobic *i*Bu side chains or blocks are not involved in the DHP binding. Therefore, the DHP uptake was likely a result of the intrinsic hydrophobicity of polymer backbones as postulated for H44 above. In the literature, amphiphilic polymers are reported to show the DHP uptake by the formation of aggregates due to the association of hydrophobic side chains [22,23]. However, the reported polymers generally have strong hydrophobic moieties such as long alkyl chains and/or higher molecular weights, which are likely to readily form hydrophobic domains in water. However, our copolymers used in this study are relatively short (DP ~40), and the *i*Bu group is relatively small, so that these copolymers may not be able to form strong hydrophobic domains. Instead, the intrinsic hydrophobicity of the polymer backbone is likely to play a more dominant role in the DHP uptake. Therefore, the observed C_DHP_ values may not present the critical concentration for the formation of polymer aggregates. Taken together, the results of the DPH uptake experiments suggest that the *i*Bu side chains or blocks do not form strong microscopic hydrophobic domains. In addition, the results also indicate that the polymer aggregates previously observed by SLS and DLS are not conventional aggregates formed by strong microscopic hydrophobic domains.

### 3.3. Cryo-TEM Observations of the Block Copolymer Aggregates

**Figure 3:**
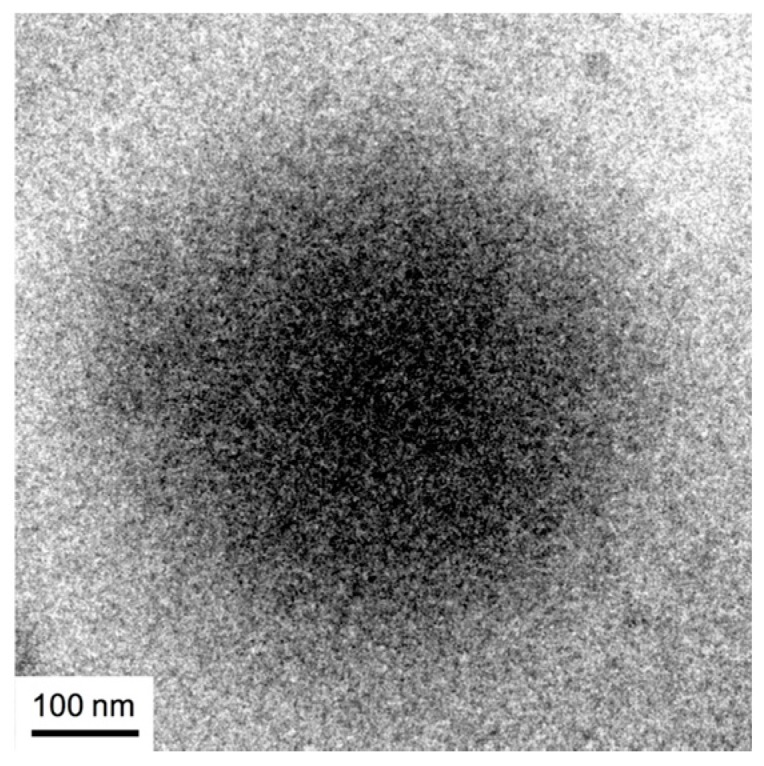
The cryo-TEM image of B38_26_ rapidly freeze-dried from 10 mg/mL solution in HEPES buffer.

The results of the DHP uptake experiments indicated that the hydrophobicity of the PIBVE blocks of B38_26_ is not sufficient for the DHP uptakes. However, our previous study demonstrated that the B38_26_ polymer chains were able to form large aggregates with diameters of 400–500 nm. To investigate the aggregation mechanism of B38_26_, we examined the structure of the aggregates at 10 mg/mL, which is substantially higher than the critical concentration observed in the DPH uptake experiments using cryo-TEM (Figure 3). The aggregate particle in the cryo-TEM image presented as a spherical blur shadow with no clear boundaries. The diameter of the particle was found to be around 500 nm, which is consistent with the results of the SLS and DLS measurements (Table 1). In our previous study, the SLS data suggested that the density of the polymer chains in the B38_26_ aggregates was relatively low, and the aggregates were relatively large, such that we speculated that B38_26_ formed a vesicle (polymer bilayers). However, the aggregate structure presented in the cryo-TEM image does not appear to have any polymer bilayers, but seems rather to consist of low-density polymer aggregates without any defined structures. 

Recently, Takahashi et al. demonstrated both experimentally [24,25,26] and theoretically [27] that if the amphiphilicity of a block copolymer is not strong enough, the copolymer does not form micelles; rather, a liquid-liquid phase separation takes place in the solution. The amphiphilicity of B38_26_ may be too weak to form micelles, and the large aggregate of a 500-nm diameter may be colloidal droplets of the phase-separated concentrated phase. If the concentration of the concentrated phase is not high, the droplet will contain a considerable amount of water, which prevents DPH uptake, and thus the contrast between the concentrated and dilute phases may be so weak that the cryo-TEM image may be blurred.

### 3.4. Fluorescnt Study of Block Copolymer Aggregates

We further investigated the formation of B38_26_ aggregates and interaction with bacteria using fluorescence spectroscopy. Here, the block copolymer B38_26_ was labeled with FITC (FITC-labeled B38_26_: F-B38_26_) [15]. The molar absorbance coefficient of F-B38_26_ was 37,000 M^−1^ cm^−1^ in HEPES buffer. Based on the molar absorbance coefficient of F-B38_26_ and the free fluorescein (83,000 M^−1^ cm^−1^), the average number of FITC molecule per B38_26_ chain was estimated to be 0.45, assuming no significant difference in the absorbance of fluorescein before and after FITC conjugation.

**Figure 4:**
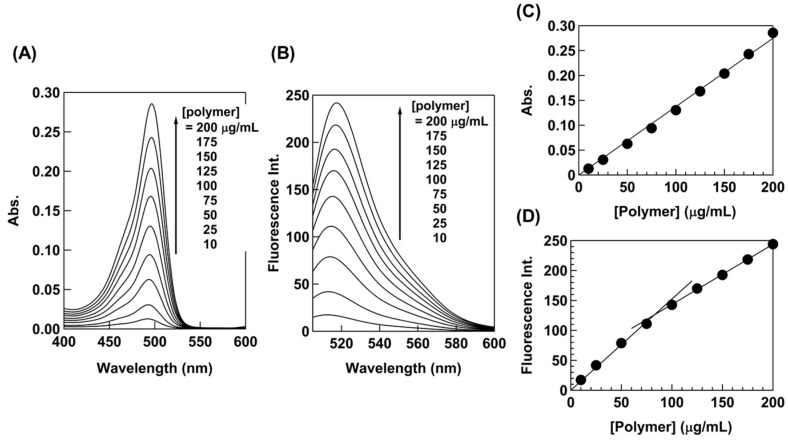
(**A**) Absorption and (**B**) emission spectra of B38_26_ containing F-B38_26_ in HEPES (1% DMSO), and (**C**) the maximum absorbance and (**D**) maximum fluorescent intensity versus polymer concentration.

First, we investigated the concentration dependence of fluorescence emission from F-B38_26_. A small amount of F-B38_26_ was added to non-labeled B38_26_ with in HEPES buffer. Based on the absorbance of 20 µg/mL polymer solution and free fluorescein absorbance, the FITC content in this mixture was estimated to be 5.3 mol % or 5.3 FITC in 100 polymer chains. The fluorescence intensity increased proportionally as a function of polymer concentration, and it exhibited a flexion point at 83 µg/mL, which may indicate that the surrounding environment of FITC in polymer chains might be changed, whereas the maximum absorbance was almost insensitive to changes in polymer concentration (Figure 4). This might reflect the onset of the formation of polymer aggregates, which change the polymer conformation and density as compared to the polymer chains free in solution.

**Figure 5:**
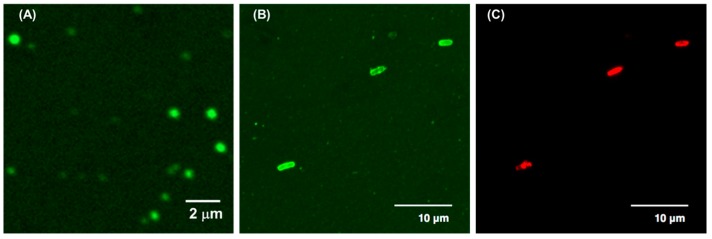
Confocal fluorescent microscopic images of (**A**) 50 µg/mL solution of B38_26_ containing F-B38_26_ (FITC: 5.3 mol %) in HEPES buffer, (**B**) *E. coli* (OD_600_ ~ 0.05) incubated with 100 µg/mL solutions of B38_26_ containing F-B38_26_ and (**C**) PI (1.6 µM) in HEPES buffer (0.5% DMSO). The images are projected images of 42 image stacks acquired with a z-step of 0.1 µm (total height: 4.2 µm).

Finally, we examined the interaction between the polymer aggregates and bacterial cells. The *E*. *coli* cells were incubated with B38_26_ containing a small amount of F-B38_26_ at 100 µg/mL, which was a higher concentration than CAC. We previously demonstrated that the polymer aggregates can be seen in fluorescence images as fluorescent particles with ~500 nm in diameter (Figure 5A) [15], which is close to the aggregate size estimated by DLS (*R*_H_ = 250 nm). The perimeters of *E. coli* cells treated with F-B38_26_ were fluorescent green, indicating the binding of the polymer on the cell surfaces (Figure 5B). However, the resolution of the images was not sufficient to identify the structure of the polymer aggregates bound on the bacterial cell surfaces. The *E. coli* cells were also stained by PI, which can only penetrate cells with damaged membranes, and shows red fluorescence [28]. The *E. coli* cells bound with F-B38_26_ showed red fluorescence, indicating that the cell membranes were damaged (Figure 5C). Liu et al. speculated that cationic polymer nanoparticles with a diameter of 177 nm caused steric hindrance and crosslinking of peptideglycans in the cell wall, disrupting cell membranes and cell death [29]. The cationic particles reported here are relatively large (400–500 nm of diameter), so the aggregates may not be able to penetrate into the cell wall structure. However, we have previously demonstrated that the BC_99.9_ values are smaller than the CAC values, suggesting that the free single polymer chain could be responsible for the bactericidal activity. Therefore, although the polymer aggregates may not be directly active against bacterial cell membranes, the polymer chains may dissociate from the polymer aggregates, and the free polymer chains may penetrate the cell wall and disrupt bacterial cell membranes to kill bacteria. The polymer aggregates are likely to have a high net-positive charge, which would facilitate the binding of aggregates onto anionic bacterial cell surfaces. The results of DPH uptake experiments and cryo-TEM observations indicate that the polymers may weakly associate to form aggregates or colloidal droplets. Therefore, the polymer chains may be able to readily dissociate to attack bacterial cell membranes after the aggregates bind to bacterial cell surfaces. The polymer aggregates may serve as a reservoir that can deliver active polymer chains to the bacterial cell surface and release them for antimicrobial actions. Our previous computational model of cationic amphiphilic methacrylate copolymers also demonstrated that the copolymer formed aggregates in an aqueous environment, but the aggregate dissociated to individual polymer chains upon binding to bacterial cell membranes [30]. Then, the free polymer chains bound to the bacterial cell membrane for antimicrobial action. These previous data also support the new perception of polymer aggregates as a delivery reservoir proposed in this study. 

## 4. Conclusions

In this study, we studied the aggregation behaviors of amphiphilic poly(vinyl ether)s with antimicrobial activity using fluorescent dye, DPH uptake assay, and fluorescent microscopy. The results of the DPH uptake experiments indicated that the hydrophobic side chains of our polymers may not form microscopic strong hydrophobic domains. The cryo-TEM images also indicated that the polymer aggregate of B38_26_ appears to have a low density of polymer chains without any defined microscopic structures. We speculate that the block copolymer, B38_26_, formed large aggregates by liquid-liquid separation due to the weak association of polymer chains, rather than the conventional core-shell type micelles or vesicles. The fluorescence microcopy images showed that B38_26_ bounds to *E. coli* cell surfaces although it was not clear that the structure of aggregates remained when bound on the cell surface. The *E. coli* cells with B38_26_ were stained by PI, indicating that the cell membranes were significantly damaged. These results suggest that the polymer aggregates may act directly by disrupting bacterial cell membranes. It is also possible that the polymer aggregates may not act directly, but that free polymer chains released from the aggregates may attack the bacterial cell membranes.

This study showed the discrepancy between methods for determining the CACs of copolymers. The CAC values determined by different methods are likely to reflect different dimensions and molecular processes (microscopic hydrophobic domains, polymer chain association, and particle formation) in the formation of polymer aggregates. It would be a subject for a future study to link the CAC values to the aggregation mechanism using different methods and determine the cause of the discrepancies. The expected results would also shed light into the polymer aggregate structures and dynamics, which would be useful for designing new antimicrobial polymer aggregates. 

Many polymer platforms have been studied, including random and block copolymers, star-shaped polymers, and graft copolymers [15,31,32]. However, the role of aggregates in their antimicrobial mechanisms is not clear yet. The physicochemical properties (charge density, size, etc.) and dynamics (exchange between polymer chains and aggregates) of polymer aggregates are likely to control the interactions with bacterial cell membranes, thus determining their antimicrobial activity. In particular, this study proposes the role of polymer aggregates as a delivery reservoir for antimicrobial action. Such properties and dynamics of polymer aggregates can be tuned by chemical compositions and structures of polymer chains. Therefore, block copolymers may provide a new programmable platform to design and develop antimicrobial materials that can utilize assembled structures and properties.

## Figures and Tables

**Table 1:** Characterization, bactericidal activity and hydrophobic dye uptake behaviors for poly(IBVE-*co*-AEVE)s.

Polymer	Copolymer Structure	DP ^1^	MP_IBVE_^1^ (mol %)	BC_99.9_^2^ (μg/mL)	HC_50_ (μg/mL)	C_DPH_^4^ (μg/mL)	CAC ^5^ (μg/mL)	*R*_H_^6^, *R*_g_^7^ (nm)
H44	Homopolymer	44	0	1.6 ± 0.0	>1000 (42.5 ± 6.3%) ^3^	90	N.D.	N.D.
B38_26_	Block copolymer	38	26	2.4 ± 0.91	>1000 (37.7 ± 2.8%) ^3^	124	36	250 ^6^
R40_25_	Random copolymer	40	25	1.6 ± 0.0	0.49 ± 0.17	125	380	27 ^7^

^1^ See [15]; ^2^ Determined in HEPES buffer against *E. coli*; ^3^ Local minimum values of hemolysis induced by each polymer; ^4^ Determined by dye uptake experiment in HEPES buffer; ^5^ Critical (intermolecular) aggregation concentration, determined by SLS; ^6^ Hydrodynamic radius, determined by DLS; ^7^ Radius of gyration, determined by SLS.

## Supplementary Materials

The following are available online at www.mdpi.com/2073-4360/10/1/93/s1, Table S1: Characterization, bactericidal activity and hydrophobic dye uptake behaviors for poly(IBVE-*co*-AEVE)s with different MP_IBVE_s, Figure S1: Fluorescence intensity of DPH (50 nM) versus polymer concentrations for poly(IBVE-*co*-AEVE)s with different MP_IBVE_s.
