# Supplementary material for: Aggregation of Cationic Amphiphilic Block and Random Copoly(vinyl ether)s with Antimicrobial Activity
Source: Polymers (Basel). 2018 Jan 19;10(1):93. doi: 10.3390/polym10010093 (PMC6414987; doi:10.3390/polym10010093)
Supplement: Supplementary file 1 [file polymers-10-00093-s001.pdf]

Supplementary Materials for

Aggregation of Cationic Amphiphilic Block and Random Copoly(vinyl ether)s with Antimicrobial Activity

Yukari Oda [1,2*], Kazuma Yasuhara [3], Shokyoku Kanaoka [1,4], Takahiro Sato [1], Sadahito Aoshima [1*], and Kenichi Kuroda [5*]

[1] Department of Macromolecular Science, Graduate School of Science, Osaka University, Toyonaka, Osaka 560-0043, Japan
[2] (current affiliation) Department of Applied Chemistry, Kyushu University, Motooka, Nishi-ku, Fukuoka 819-0395, Japan
[3] Graduate School of Materials Science, Nara Institute of Science and Technology, Ikoma, Nara 630-0192, Japan
[4] (current affiliation) Department of Materials Science, The University of Shiga Prefecture, Hikone, Shiga 522-8533, Japan
[5] Department of Biologic and Materials Science, School of Dentistry, University of Michigan, Ann Arbor, Michigan 48109, United States

Table S1. Characterization, bactericidal activity and hydrophobic dye uptake behaviors for poly(IBVE-*co*-AEVE)s with different MP_{IBVE}s.

Polymer	Copolymer Structure	DP[1]	MP_{IBVE}[1] (mol %)	$BC_{99.9}$[2] (μg/mL)	HC_{50} (μg/mL)	C_{DPH}[4] (μg/mL)
$B39_{51}$	Block copolymer	39	51	3.1±0.0	> 1000 (12.9±6.3%)[3]	85
$B39_{77}$		39	77	62.5±0.0	> 1000 (22.8±8.5%)[3]	80
$R38_{53}$	Random copolymer	38	53	3.1±0.0	1.8±0.2	72
$R38_{79}$		38	79	31.3±0.0	18.9±1.3	90

[1]See *Biomacromolecules* **2011**, *12*, 3581-3591.

[2]Determined in HEPES buffer against *E. coli*.

[3]Local minimum values of hemolysis induced by each polymer.

[4]Determined by dye uptake experiment in HEPES buffer.

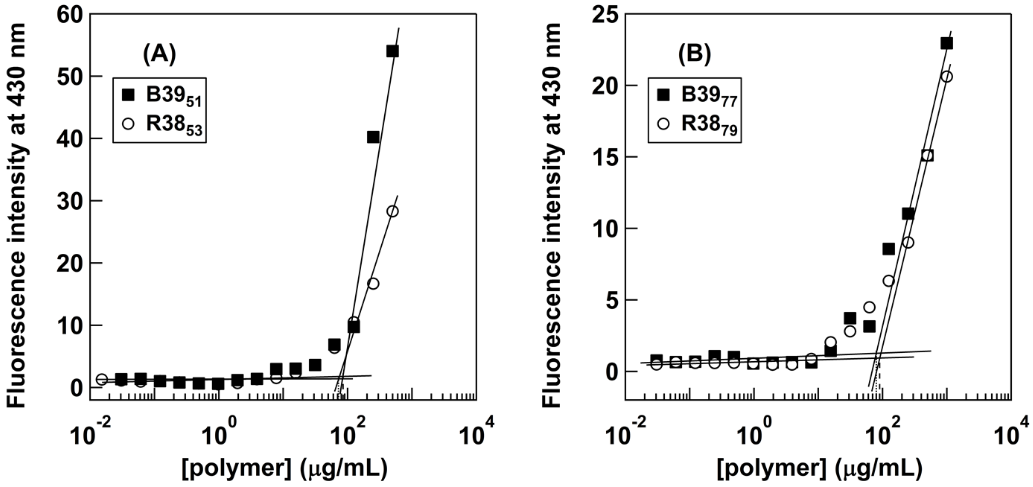

Figure S2. Fluorescence intensity of DPH (50 nM) versus polymer concentrations of (A) $B39_{51}$ and $R38_{53}$, and (B) $B39_{77}$ and $R38_{79}$. The data points represent the average from duplicate measurements.
